# Exosome derived from epigallocatechin gallate treated breast cancer cells suppresses tumor growth by inhibiting tumor-associated macrophage infiltration and M2 polarization

**DOI:** 10.1186/1471-2407-13-421

**Published:** 2013-09-17

**Authors:** Ji-Young Jang, Jong-Kuen Lee, Yoon-Kyung Jeon, Chul-Woo Kim

**Affiliations:** 1 Tumor Immunity Medical Research Center, Cancer Research Institute, Seoul National University College of Medicine, 28 Yongon-dong, Jongno-gu, Seoul 110-799, Korea; 2 Department of Pathology, Seoul National University College of Medicine, 28 Yongon-dong, Jongno-gu, Seoul 110-799, Korea

**Keywords:** EGCG, Exosomes, miR-16, Tumor microenvironment, Tumor-associated macrophages (TAM)

## Abstract

**Background:**

Tumor-associated macrophages (TAM) play an important role in tumor microenvironment. Particularly, M2 macrophages contribute to tumor progression, depending on the expression of NF-κB. Tumor-derived exosomes can modulate tumor microenvironment by transferring miRNAs to immune cells. Epigallocatechin gallate (EGCG) has well known anti-tumor effects; however, no data are available on the influence of EGCG on communication with cancer cells and TAM.

**Methods:**

Murine breast cancer cell lines, 4T1, was used for *in vivo* and *ex vivo* studies. Exosome was extracted from EGCG-treated 4T1 cells, and the change of miRNAs was screened using microarray. Tumor cells or TAM isolated from murine tumor graft were incubated with exosomes derived from EGCG-treated and/or miR-16 inhibitor-transfected 4T1 cells. Chemokines for monocytes (CSF-1 and CCL-2), cytokines both with high (IL-6 and TGF-β) and low (TNF-α) expression in M2 macrophages, and molecules in NF-κB pathway (IKKα and Iκ-B) were evaluated by RT-qPCR or western blot.

**Results:**

EGCG suppressed tumor growth in murine breast cancer model, which was associated with decreased TAM and M2 macrophage infiltration. Expression of chemokine for monocytes (CSF-1 and CCL-2) were low in tumor cells from EGCG-treated mice, and cytokines of TAM was skewed from M2- into M1-like phenotype by EGCG as evidenced by decreased IL-6 and TGF-β and increased TNF-α. *Ex vivo* incubation of isolated tumor cells with EGCG inhibited the CSF-1 and CCL-2 expression. *Ex vivo* incubation of TAM with exosomes from EGCG-treated 4T1 cells led to IKKα suppression and concomitant I-κB accumulation; increase of IL-6 and TGF-β; and, decrease of TNF-α. EGCG up-regulated miR-16 in 4T1 cells and in the exosomes. Treatment of tumor cells or TAM with exosomes derived from EGCG-treated and miR-16-knock-downed 4T1 cells restored the above effects on chemokines, cytokines, and NF-κB pathway elicited by EGCG-treated exosomes.

**Conclusions:**

Our data demonstrate that EGCG up-regulates miR-16 in tumor cells, which can be transferred to TAM via exosomes and inhibits TAM infiltration and M2 polarization. We suggest a novel mechanism by which EGCG exerts anti-tumor activity via regulation of TAM in tumor microenvironment.

## Background

Worldwide, breast cancer is the most frequently diagnosed life-threatening cancer and the most important cause of cancer-related deaths in women [1]. Population-based investigations have suggested dietary factors which affect the risk of breast cancer [2,3]. Epidemiological studies of Asian and Chinese women have reported an inverse association between the consumption of green tea and the risk of breast cancer. Of the polyphenols present in green tea, epigallocatechin-3-gallate (EGCG) has been identified as having inhibitory effects on tumorigenesis in studies using *in vitro* and *in vivo* models of carcinogenesis [4-7]. Anti-tumorigenic activities of EGCG include inhibition of cell proliferation, induction of apoptosis and cell cycle arrest, inhibition of invasion and metastasis, and suppression of angiogenesis [8-13].

Exosomes are circular fragments of membrane released from the endosomal compartment, and are shed from the surface membranes of most cell types [14,15]. An increasing body of evidence indicates that exosomes play a pivotal role in cell-to-cell communication [16], and in particular, tumor cells are found to release large quantities of exosomes [17-19]. The amount of circulating exosomes is greater in the serum or plasma of patients with cancer and predict a poor prognosis [17]. Release of exosomes may protect tumor cells from apoptosis by selective extrusion of apoptosis-inducing proteins. Additionally, exosomes may help tumor cells escape the immune surveillance [18] and carry out pro-angiogenic signals that increase tumor vascularization [20]. In addition, exosomes may transfer genetic information, such as microRNAs (miRNAs) from tumor cells to neighboring cells [21].

Macrophages populate the microenvironment of most tumors. In certain cases, these cells can represent more than half of the tumor mass and play an important role in tumor immunity, which is particularly true for breast cancer [22]. Clinical studies have sought to correlate macrophage density and cancer prognosis. A meta-analysis have shown that, in 80% of the cases, increased macrophage density was associated with poor prognosis, and that, in the remaining 20%, there was a split between null prognostic value and good prognostic value [23]. Studies of this nature have been performed most extensively for breast cancer, and multiple independent investigations have found increased quantities of tumor-associated macrophages (TAMs) to be associated with poor prognosis [24]. In addition to the extent of macrophage infiltration, the phenotype of TAMs has been shown to affect tumor progression [25]. Within the tumor microenvironment, several stimuli are known to influence the TAM phenotype. Macrophages can be induced to either tumor-suppressive immunological type (referred to as M1) or tumor-promoting inflammatory/immune-suppressive population (M2 macrophages) [26,27]. Tumor cells produce colony-stimulating factor-1 (CSF-1) and Chemokine (C-C motif) ligand 2 (CCL2), which are two major attractants and growth factors for TAM. The concept that TAM are mainly M2 activated, or even M2 ‘polarized’, has been around for almost a decade, and is corroborated by the pattern of TAM marker expression. High production of IL-10 and low production of IL-12 is seen as a hallmark of all non-M1 macrophages, and is also applicable to most TAM populations in different cancer types. Accordingly, high frequency of infiltrating TAM is associated with a poor prognosis for many types of tumors. This pathological association to clinical progression has reemerged in the post-genomic era: genes associated to macrophage infiltration are the same molecular signatures that herald poor prognosis in lymphomas and breast carcinoma patients [28].

We hypothesized that EGCG might regulate the expression of tumor-derived exosomal miRNAs and affect the tumor microenvironment and TAMs. The aim of this study was to investigate the effect that EGCG has on tumor-derived exosomal miRNAs and TAM.

## Methods

### Cell lines and reagents

The mouse mammary tumor cell line, 4T1, were maintained as monolayer cultures in Dulbecco’s Modified Eagle Medium (DMEM), supplemented with 10% fetal bovine serum (FBS), 100 units/ml penicillin, and 100 μg/ml streptomycin (Gibco, Grand Island, NY, USA) in a humidified 5% CO_2_/95% air atmosphere at 37°C. The murine RAW264.7 macrophage cell line were grown in RPMI 1640 containing 10% fetal bovine serum 100 units/ml penicillin, and 100 μg/ml streptomycin (Gibco, Grand Island, NY, USA) in a humidified 5% CO_2_/95% air atmosphere at 37°C. Epigallocatechin gallate (EGCG) and lipopolysaccharides (LPS) were purchased from Sigma (St. Louis, MO, USA).

### Transfection

The mouse mammary tumor cell line, 4T1 or macrophages cell line, RAW264.7 were plated on six-well plates (2 × 10^5^ cells per well) and were allowed to adhere for 24 hours. These cells were transfected with either scramble miRNA inhibitor or miR-16 inhibitor using Lipofectamine 2000 (Invitrogen, Carlsbad, CA). Transfected cells were then cultured for 6 hours, and culture media were replaced with fresh media supplemented with 10% FBS. The cells were harvested at 24–48 hours after transfection. The scramble miRNA inhibitor or miR-16 inhibitor (100 nM) were obtained from Shanghai GenePharma Co (Shanghai, China). The scramble miRNA mimics or miR-16 mimics (100 nM) were obtained from Genolution (Seoul, Korea).

### Exosome*s* isolation and purification

The 4T1 mouse mammary tumor cells (or TAM cells) were centrifuged overnight at 100,000 g to isolate bovine-derived exosomes which are present in the DMEM. The exosomes from 4T1 cells were isolated from the remaining supernatants using ExoQuick (System Bioscience, Mountain View, CA, USA) according to the manufacturer’s protocol. The pellets were washed in large volumes of PBS and resuspended in 80 μl PBS. Proteins in pellets and lysates were quantified by Micro-BCA (Thermo Scientific) in the presence of 2% SDS. Purity of isolated exosome was assured using electron microscopy by exosomal size or immunobloting for CD63, tsg101, and calnexin.

### Quantification of miR-16 by RT-qPCR

The 4T1 mammary tumor cells were grown in 6 cm Petri dishes to 70% confluence then were treated for 24 hr with 100 *μ*M EGCG. Total RNA was extracted from these cells using Trizol reagent (Invitrogen). The RNA quality, yield, and size of extracted RNA were analyzed using capillary electrophoresis (Agilent 2100 Bioanalyzer, Agilent Technologies, Foster City, CA, USA). RT-qPCR analysis for miRNAs was carried out with Mir-X™ miRNA First-Strand Synthesis Kit (Clontech, CA, USA) and SYBR Green Real time PCR Master Mix (TaKaRa, Otsu, Japan) according to the respective manufacturer’s instruction. The house-keeping (HK) gene U6 was used as a control for standardization of the initial miRNA content of a sample. Relative changes of gene expression were calculated by the following formula, and the data was represented as fold up-regulation/down-regulation, fold change = 2^-ΔΔCt^, where ΔΔCt = (Ct of gene of interest, treated -Ct of HK gene, treated)-(Ct of gene of interest, control-Ct of HK gene, control). The primers used were as follows: for miR-16, forward 5′-TAGCAGCACGTAAATATTGGCG-3′; for U6, forward 5′-TGGCCCCTGCGCAAGGATG-3, and miR-16 and U6 reverse primer was included in Mir-X™ miRNA First-Strand Synthesis Kit.

### Prediction of miRNA targets

Web resources was used to predict miR-16 targets, including a viewer for browsing potential target sites, conserved with or without positional constraints, on aligned UTRs, with periodic updates (http://www.microrna.org).

### Quantitative real-time RT-PCR (RT-qPCR)

Total RNA was extracted using Trizol (Invitrogen). For RT-qPCR analysis, 5 μg of RNA was reverse-transcribed using RT-PCR kits (Promega, Madison, WI, USA). PCR was performed using SYBR Green Real time PCR Master Mix (TaKaRa, Otsu, Japan). Relative changes of gene expression were calculated by the following formula:

ΔΔCt = (Ct of gene of interest, treated -Ct of HK gene, treated)-(Ct of gene of interest, control-Ct of HK gene, control); where, Ct was the threshold cycle number, and HK was the house keeping gene.

This data was represented as fold up-regulation/down-regulation, fold change = 2^-ΔΔCt^. The primer sequences were used as follows: for TGF-β, forward sequence 5′- CACCGGAGAGCCCTGGATA-3′ reverse sequence 5′- TGTACAGCTGCCGCACACA-3′; IL-6 forward sequence 5′-GACAACTTTGGCATTGTGG-3′ reverse sequence 5′-ATGCAGGGATGATGTTCTG-3′; TNF-α forward sequence 5′-TCCCAGGTTCTCTTCAAGGGA-3′ reverse sequence 5′-GGTGAGGAGCACGTAGTCGG-3′; CSF-1 forward sequence 5′-CGACATGGCTGGGCTCCC-3′ reverse sequence 5′- CGCATGGTCTCATCTATTAT-3′; CCL-2 forward sequence 5′-GTTGGCTCAGCCAGATGCA-3′ reverse sequence 5′-AGCCTACTCATTGGGATCATCTTG-3′; IL-1β, forward sequence 5′-AAGGAGAACCAAGCAACGACAAAA-3′ reverse sequence 5′-TGGGGAACTCTGCAGACTCAAACT-3′.; GAPDH forward sequence 5′- GGGCTGGCATTGCTCTCA-3′ reverse sequence 5′- TGCTGTAGCCGTATTCATTGT-3′.

### Western blotting

For western blot analyses, the cells were harvested after 24 hours of exosomes treatment and lysed with lysis buffer (5 mM/L ethylenediamine tetra acetic acid; 300 mM/L NaCl; 0.1% NP-40; 0.5 mM/L NaF; 0.5 mM/L Na3VO4; 0.5 mM/L phenylmethylsulfonyl fluoride; and 10 μg/ml each of aprotinin, pepstatin, and leupeptin; Sigma, St Louis, MO). Following centrifugation at 15,000 g for 30 minutes, the concentrations of supernatant proteins were analyzed using the Bradford reagent (Bio-Rad, Hercules, CA). For analysis of protein contents, 50 μg of total proteins was electrophoresed in 10% SDS-PAGE gel, transferred to polyvinylidene difluoride membranes (Millipore, Bedford, MA), and were incubated with antibodies against IKKα(Abcam), I-κB (Santa Cruz Biotechnology), IL-6 (Invitrogen : AMC0864), TNF-α (Abcam : ab1793), TGF-β (Abcam : ab64715) or actin (Sigma). Immunoblots were visualized using an enhanced chemiluminescence detection system (Amersham Pharmacia Biotech, Uppsala, Sweden).

### Tumor-associated macrophages isolation from tumor tissue

For *ex vivo* assay, the 4T1 cells (1 × 10^5^) were suspended in 100 μl PBS and then injected subcutaneously into either side of the posterior flank of six BALB/c mice to induce tumor growth. When these cells have established as tumors after 3-to-4 weeks, the resulting tumor mass were harvested for isolation of TAMs. Each tumor tissue was cut into 2 mm fragments, followed by collagenase digestion (0.3 mg/ml, Worthington Biochemical Corp, NJ, USA) for 1 h at 37°C. The suspension was filtered through a 70-μm stainless steel wire mesh to generate a single-cell suspension. This resulting suspension was centrifuged and washed twice with PBS (pH = 7.4). CD11b myeloid cells were purified from tumor cell suspension using the MACS method (Militenyi Biotec). Briefly, the CD11b cells were incubated with beads conjugated with anti-mouse CD11b and were positively selected on LS columns. The purity of recovered cells assessed by flow cytometry was greater than 95%. The viability of isolated cells routinely exceeded 90%, as determined by trypan blue exclusion assays. These TAMs (5 × 10^5^/ml) were stimulated with the exosomes (50 μg/ml) from the part of the experiment describe above.

### *In vivo* study

For *in vivo* assay, the 4T1 cells (1 × 10^5^) were suspended in 100 μl PBS and then injected subcutaneously into either side of the posterior flank of six BALB/c mice. Tumor growth was examined daily, and the tumor volumes were calculated every week using the formula for hemi-ellipsoids: *V* = length (cm) × width (cm) × height (cm) × 0.5236. After 5 weeks, each mouse was sacrificed, and the tumors were dissected and weighed. Animals experiment for this research was designed and carried out according to the standard guideline of Institutional Animal Care and Use Committee (IACUC), and the study design had been approved by Seoul National University Institutional Animal Care and Use Committee (http://iacuc.snu.ac.kr).

### Immunohistochemistry

Tumor tissue was fixed in 4% buffered neutralized formalin for 48 hours. After embedding in paraffin, 4 μm serial sections were made and mounted on a slide. Phenotypic characterization of macrophages was performed using double immunohistochemical staining to enable evaluation of tumor cells. Antigens were retrieved by boiling tissue sections in citrate buffer (DakoCytomation, Glostrup, Denmark) for 10 minutes. Anti-mouse macrophage CD68 mAb (ab31630; Abcam, Cambridge, UK) was used as a marker for all macrophages, and anti-mouse CD163 mAb (NBP1-95135; Novus, Missouri, USA) as a marker for M2-type macrophages.

### Statistical analysis

We employed GraphPad Prism software to conduct statistical analysis. Results are expressed as the mean ± s.e.m. For p-value calculation, unpaired Student's *t* tests (two tailed) were utilized for all comparisons. Null hypotheses of no difference were rejected if p-values were less than 0.05.

## Results

### EGCG suppresses tumor growth, macrophages infiltration, and M2 polarization in *in vivo* murine breast cancer model

**Figure 1:**
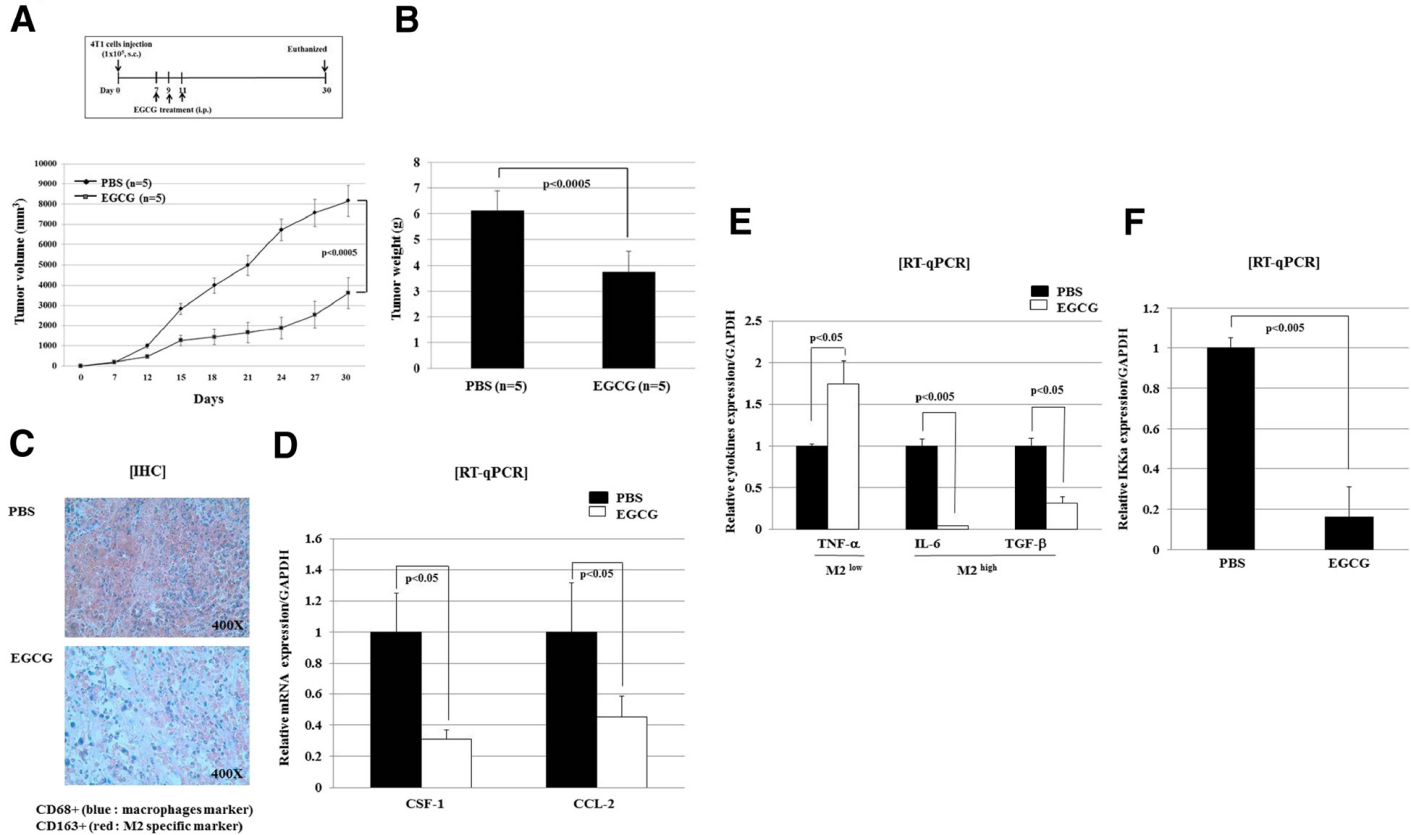
**EGCG suppresses tumor growth, macrophages infiltration, and M2 polarization in *in vivo *murine breast cancer model. (A)** Balb/c mice were challenged with 1× 10^5^ murine breast cancer cell lines, 4T1, by subcutaneous injection into the right flanks. EGCG (10 mg/kg) or PBS (as a negative control) was administrated intraperitoneally (i.p.) on days 7, 9, and 11. Tumour sizes were measured every week until day 30 post tumour challenge. **(B)** Mice were sacrificed and the tumors were dissected and the weight was measured. **(C)** For evaluation of tumor-associated macrophages and M2 macrophage infiltration, immunohistochemical staining for CD68 and CD163 was done using tumor tissue. **(D)** Tumor cells were isolated and total RNA was extracted and then subjected to RT-qPCR to measure the levels of CSF-1, CCL-2, and GAPDH. Histogram shows the relative expression of molecules compared to GAPDH as internal control. **(E)** Tumor-associated macrophages were isolated, and total RNA from these cells were extracted and subjected to RT-qPCR to detect levels of M2^high^ cytokines (IL-6 and TGF-β) and M2^low^ cytokine (TNF-α). Histogram shows the relative expression of molecules compared to GAPDH as internal control. **(F)** Tumor-associated macrophages were isolated and total RNA from cells were extracted and then subjected to RT-qPCR to detect the level of IKKα and GAPDH (internal control).

To determine if the presence of EGCG has influenced tumor growth, TAM infiltration and differentiation, we established a murine tumor model by injecting murine breast cancer cell lines, 4T1, subcutaneously into syngeneic mice. Mice were then treated with EGCG either PBS as a control by intraperitoneal injection as described in Figure 1A. At 30 days after tumor challenge, a significant decrease of tumor volume and weight was observed in the EGCG-treated group versus the control group (p < 0.0005) (Figure 1A and 1B). Intra-tumoral infiltrations of TMA and M2 macrophages as assessed by immunohistochemical staining were lower in EGCG-treated group than control (Figure 1C).

We next measured the levels of CSF-1 and CCL-2, major chemokines involved in the monocyte recruitment into tumor microenvironment [29], in tumor cells isolated from mice by RT-qPCR. In line with the decreased number of TAM in EGCG-treated mice, expressions of both CSF-1 and CCL-2 were significantly decreased upon EGCG treatment (Figure 1D). Differentiation of TAM into pro-tumoral M2 macrophages is induced by a set of cytokines including IL-6 and TGF-β, and M2 macrophages produce much lower levels of TNF-α, compared to M1 macrophages. Therefore, we isolated TAM from mice and evaluated the expression of these cytokines by RT-qPCR. TAM from tumors treated with EGCG showed significant decrease of IL-6 and TGF-β level, and higher expression of TNF-α compared with those TAMs from control group (Figure 1E). NF-κB activation in macrophages within tumor microenvironment has been known to differentiate TAM to pro-tumoral and immunosuppressive M2 phenotype during the early stage of tumor development [30]. Because IKKα has a central role for mediating canonical NF-κB activation pathway, we checked the level of IKKα in TAM from mice by RT-qPCR and found that IKKα mRNA level was significantly decreased in TAM of EGCG-treated group compared with control group (Figure 1F).

Altogether these data suggested that EGCG treatment suppresses *in vivo* TAM infiltration and inhibits polarization of TAM into tumor-promoting M2 macrophages, which was involved by decreased production of chemo-attractants, CSF-1 and CCL-2, from tumor cells, and down-regulation of M2 macrophage-associated cytokines, IL-6 and TGF-β and relative up-regulation of M1 macrophage-associated cytokine, TNF-α, in TMA from EGCG-treated tumor. Inhibition of NF-κB activity of TAM by EGCG treatment might be implicated in these processes. Importantly, our result suggested that EGCG might exert anti-tumor activity by preventing TAM from achieving pro-tumoral properties in tumor microenvironment.

### Exosomes derived from EGCG-treated tumor cells leads to decrease of CSF-1 and CCL-2 expression in a paracrine manner, and suppresses M2 polarization of TAM in *ex vivo* study

**Figure 2:**
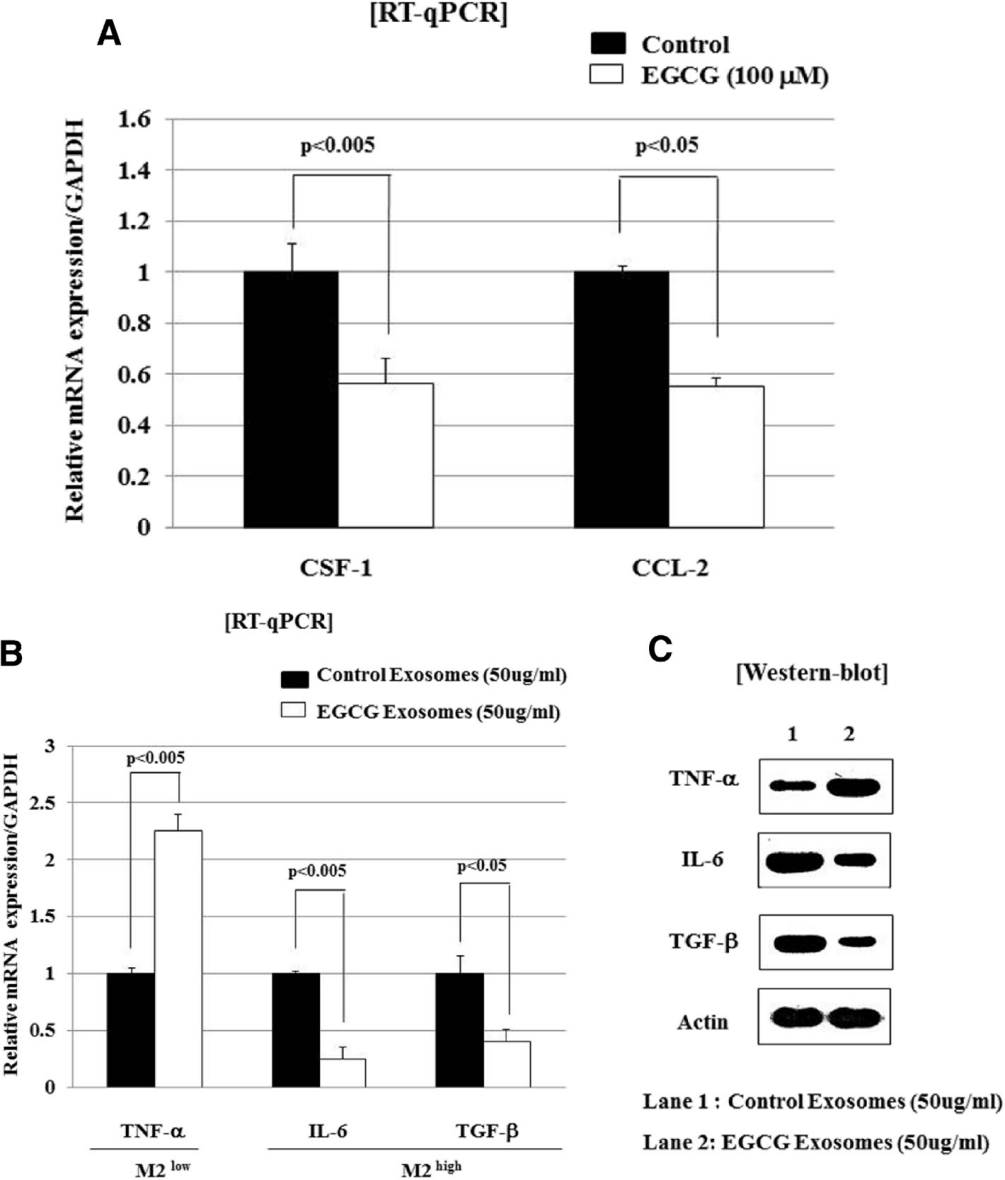
**Exosomes-derived from EGCG-treated tumor cells down-regulate chemo-attractants in tumor cells, and suppress M2 polarization of tumor-associated macrophages in *ex vivo *model. (A)** Tumor cells were isolated from established murine breast cancer using 4T1 cell line as in Figure 1, and were treated with EGCG (100 μM). After 24 hours of incubation, total RNA from cells were extracted and subjected to RT-qPCR to detect the levels of CSF-1, CCL-2, and GAPDH. Histogram shows the relative expression of molecules compared to GAPDH as internal control. **(B)** Tumor-associated macrophages were isolated from established murine breast cancer using 4T1 cell lines, and were treated with exosomes (50 μg/ml) derived from EGCG-treated or untreated 4T1 cells. After 24 hours of incubation, total RNA from cells were extracted and subjected to RT-qPCR to evaluate the levels of M2^high^ cytokines (IL-6 and TGF-β) and M2^low^ cytokine (TNF-α). Histogram shows the relative expression of molecules compared to GAPDH as internal control. **(C)** Tumor-associated macrophages were isolated from established murine breast cancer cell lines, and treated with exosomes (50 μg/ml) derived from EGCG-treated or untreated 4T1 cells. After 24 hours of incubation, total lysates from cells were extracted and subjected to Western-blot to evaluate the protein levels.

Tumor cells release chemo-attractants to recruit and activate macrophages in a paracrine loop between tumors and macrophages, and this release promotes breast cancer invasion [31]. Exosomes could play an important role of communication between tumor cells and macrophages. In order to determine whether the effect of EGCG observed in the *in vivo* mouse model could be mediated by tumor exosome, we carried out the *ex vivo* experiments using the exosomes extracted from 4T1 cell lines. As shown in Figure 2A, treatment of tumor cells with EGCG resulted in a significant decrease of both CSF-1 and CCL-2 expression. In addition, *ex vivo* incubation of TAM with exosomes from EGCG-treated 4T1 cells skewed polarized these macrophages away from the tumor-promoting M2- to a tumor-inhibiting M1-like phenotype, as evidenced by down-regulated the expression of IL-6 and TGF-β, but up-regulated TNF-α expression (Figure 2B). We confirmed the protein levels of IL-6, TGF-β, and TNF-β and obtained results same as in RT-qPCR (Figure 2C). These data indicated that exosomes derived from EGCG-treated tumor cells suppress TAM infiltration and inhibit TAM differentiation into M2 macrophages in murine breast cancer model.

### EGCG up-regulates cellular and exosomal miR-16 levels in murine breast cancer cells, 4T1

To investigate the mechanism by which EGCG treatment had led to decreased TAM infiltration and inhibition of M2 polarization via exosomes, we focused on the effect of EGCG on the regulation of tumor exosomal miRNA. We hypothesized that EGCG might change the miRNA expression in tumor cells and subsequently in exosomal compartment, and that the exosomal miRNA to TAM would influence on the recruitment and differentiation of TAM. To address this issue, we first screened the miRNAs whose expressions are modulated in 4T1 cells by miRNA microarray analysis using both total cellular miRNA and exosomal miRNA after treatment with 100 μM of EGCG for 24 h. In brief, a set of miRNAs including let-7, miR-16, miR-18b, miR-20a, miR-25, miR-92, miR-93, miR-221, and miR-320 were up-regulated, and dozens of miRNAs including miR-10a, miR-18a, miR-19a, miR-26b, miR-29b, miR-34b, miR-98, miR-129, miR-181d were down-regulated in both total cellular and exosomal fraction by EGCG treatment (data not shown). Of these, miRNAs up-regulated by EGCG were suitable for further *in vitro* study because it is more likely that over-expressed miRNA could be stored within exosome and then transferred to TAM. Specifically, the miR-16 was selected because it was elevated by EGCG treatment and has been known to be associated with immune cell function.

**Figure 3:**
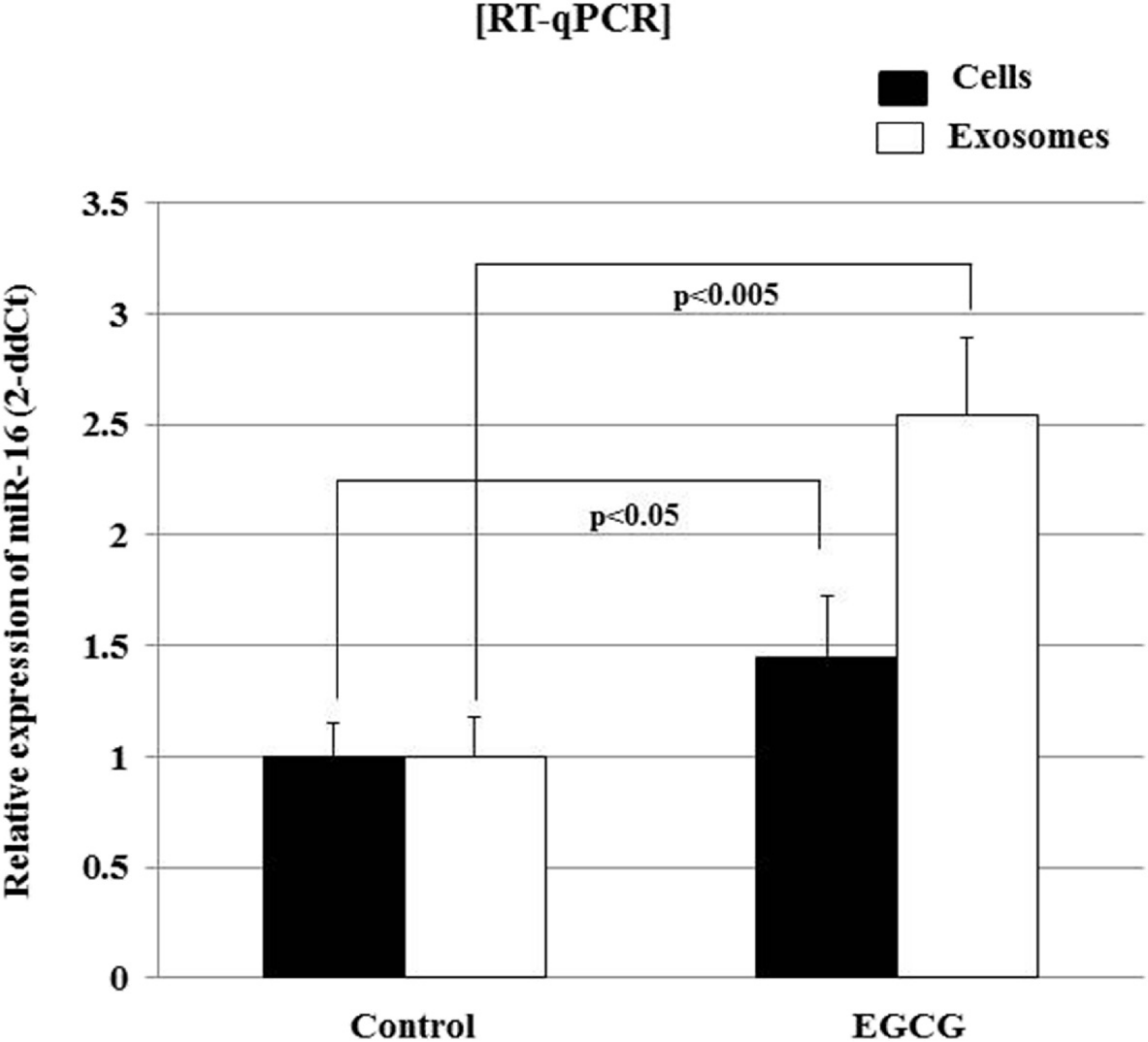
**EGCG increases cellular and exosomal miR-16 expression in murine breast cancer cell line, 4T1.** 4T1 cells were treated with and without EGCG (100 μM) for 48 hours. Then, total RNA from cells or secretory exosomes was extracted and subjected to RT-qPCR to assess the miR-16 level. Histogram shows the relative expression of miR-16 compared to U6 as an internal control.

To validate the microarray data, 4T1 cells were incubated with EGCG, and the total RNA was extracted from cells and secretory exosomes. This was then analyzed by RT-qPCR. Significant up-regulation of miR-16 in both EGCG-treated cells and exosomes was observed with a 1.45 and 2.54 folds change compared with the levels of controls (p < 0.05 and p < 0.005, respectively) (Figure 3).

### Up-regulation of exosomal miR-16 by EGCG treatment down-regulates IKKα and subsequently induces IκB accumulation in TAM, and inhibits M2 polarization

MiR-15a and miR-16 have been known to act as a negative regulator of NF-κB activity by regulating IKKα expression, which contributes to the ability of miR-15 and niR-16 as a tumor suppressor. NF-κB activation is also important for monocyte differentiation into macrophage and TAM. In fact, a study has reported that during monocyte-macrophage differentiation, expressions of miR-15a and miR-16 were decreased with higher expression of the IKKα [30].

**Figure 4:**
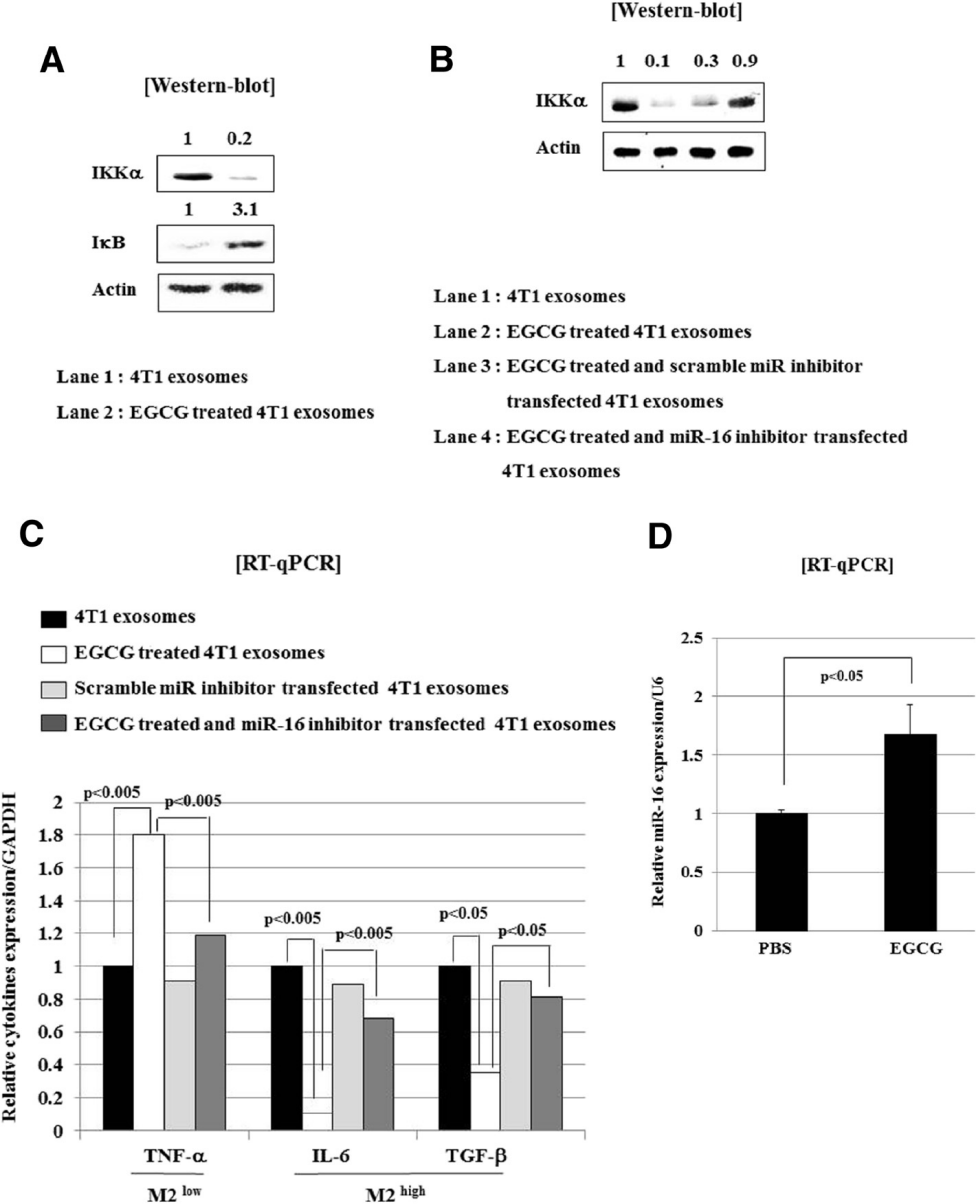
**Exosomes derived from EGCG-treated tumor cells suppresses NF-κB pathway and inhibits M2 polarization of tumor-associated macrophages, which is dependent on up-regulated miR-16 in EGCG-treated tumor exosomes. (A)** Tumor-associated macrophages were isolated from established murine breast cancer 4T1 cell lines, and treated with exosomes extracted from EGCG (100 μM)-treated and untreated 4T1 cells for 24 hours. Whole cell lysates were prepared and submitted to Western blot using anti-IKKα, anti-I-κB or anti-actin antibodies. **(B)** 4T1 cells were incubated with EGCG (100 μM) and simultaneously (24 hours) transfected with scramble or miR-16 inhibitor for 24 hours. Exosomes were then extracted from each tumor cells. Tumor-associated macrophages isolated from established murine breast cancer were treated with each exosome as indicated at the concentration of 50 μg/ml for 24 hours, and whole cell lysates were prepared and submitted to Western blot using anti-IKKα or anti-actin antibodies. The numbers in Figure 4A and 4B report the quantification of bands by imaging analysis with KODAK Molecular Imaging Software (MI). **(C)** 4T1 cells were incubated with EGCG (100 μM) and were simultaneously (24 hours) transfected with scramble or miR-16 inhibitor for 24 hours. Exosomes were then extracted from each tumor cells. Tumor-associated macrophages isolated from established murine breast cancer were treated with each exosome at the concentration 50 mg/ml for 24 hours, and total cellular RNA was extracted and submitted to RT-qPCR analysis for M2^high^ cytokines (IL-6 and TGF-β) and M2^low^ cytokine (TNF-α). Histogram shows the relative expression of molecules compared to GAPDH as internal control. **(D)** Tumor cells were isolated and total RNA from cells were extracted and subjected to RT-qPCR to measure the miR-16 level. Histogram shows the relative expression of miR-16 compared to U6 as an internal control.

As already known, we identified the possible target sequence in the 3′-UTR of IKKα for miR-16. Therefore, we tested whether exosomes derived from EGCG-treated 4T1 cells could suppress IKKα expression and, if so, whether that process occurs through exosomal miR-16. As shown in Figure 4A, the treatment of TAM with exosomes from EGCG-treated 4T1 cells has resulted in decreased IKKα protein expression and concomitant accumulation of the I-κB in TAM. Moreover, when TAM was incubated with exosomes from EGCG-treated and miR-16 inhibitor-transfected 4T1 cells, IKKα levels had recovered to the that of the control (4T1 exosome treated TAM) (Figure 4B). MiR-16 expression was not elevated in exosomes from EGCG-treated and miR-16 inhibitor-transfected 4T1 cells (data not shown), unlike exosomes from EGCG-treated 4T1 cells where miR-16 was up-regulated by EGCG (Figure 3).

To test whether exosome from EGCG-treated 4T1 cells could inhibit M2 polarization of TAM through exosomal miR-16, we treated TAM with exosomes either from EGCG-treated 4T1 cells or from from EGCG-treated and miR-16 inhibitor-transfected 4T1 cells, and measured the level of cytokines including IL-6, TGF-β, and TNF-α. Consistent with the *in vivo* data (Figure 1E), incubation of TAM with exosomes from EGCG-treated 4T1 cells had led to the suppression of M2-associated cytokines, IL-6 and TGF-β, and elevation of the M1-related cytokine, TNF-α (Figure 4C). More importantly, this alteration of cytokines was restored when TAM was incubated with exosomes from EGCG-treated and miR-16 inhibitor-transfected 4T1 cells (Figure 4C). We verified that the levels of miR-16 are reduced by transfection of miR-16 inhibitor (Additional file 1). Finally, we observed a 1.68-fold increase of miR-16 expression in tumor cells from mice treated with EGCG compared to control (Figure 4D). To investigate modulation of macrophage by miR-16, mouse macrophage cell line, RAW264.7, cells was transfected with scramble or miR-16 mimics and stimulated with 5 μg/ml LPS. We found that miR-16 mimics significantly suppressed LPS-induced IL-1β and IL-6 production in these RAW264.7 cells (Additional file 2).

**Figure 5:**
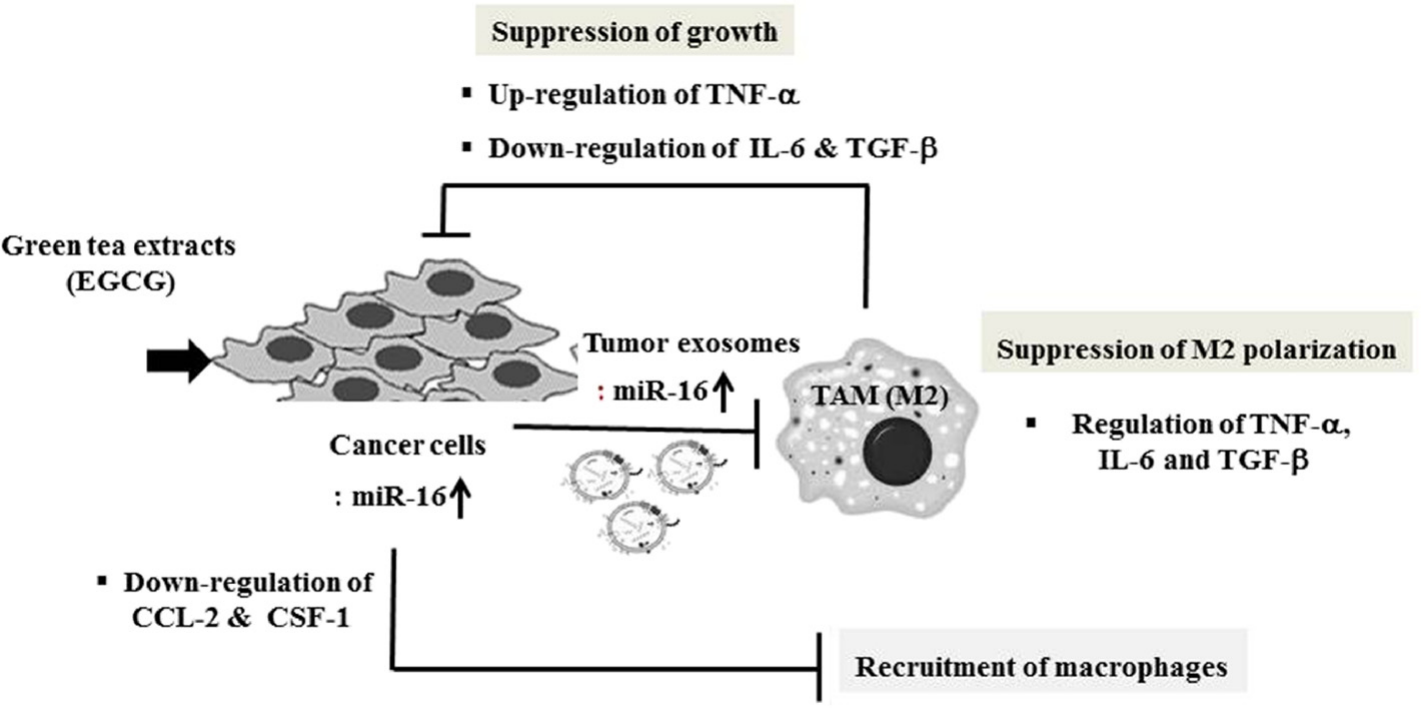
Proposed mechanisms of EGCG to inhibit infiltration of tumor-associated macrophages and M2 polarization in tumor microenvironment.

Together, the results suggest that EGCG treatment leads to up-regulation of miR-16, which might be transferred by exosomes to TAMs, and contributes to the suppression of NF-κB activity by down-regulating IKKα and subsequently accumulating Iκ-B, and inhibition of TAM infiltration and M2 polarization. These molecular pathways might represent a new mechanism by which EGCG exerts anti-tumor effect through manipulating TAM and tumor microenvironment, as illustrated in Figure 5.

## Discussion

Exosomes are vesicles with a diameter of 50–100 nm secreted from intracellular endosomes. These vesicles are unrelated to the RNA exosome, which is an RNA-processing intercellular complex. Membrane vesicles, which are also referred to as microvesicles or microparticles, have a diameter of 100–1,000 nm and are presumed to be formed by budding or shedding from plasma membrane [32]. Studies of the functional contributions of these vesicles to intercellular communication have focused on understanding the role of their membranous and cytoplasmic protein content. A role in the modulation of immune function has also emerged because the cellular microenvironment is a critical determinant of tumor progression and development [33].

There is much interest in intercellular communication between tumor cells and immune cells in the tumor microenvironment. Cells use a variety of approaches to communicate with each other, such as direct membrane-to-membrane contact or by the release of soluble mediators. Among these various means of information transfer, exosomes represent a unique way to mediate cell-to-cell communication [34-38]. Exosomes contain membrane proteins similar to those of the donor cell and contain protein and RNA derived from the donor cell cytoplasm [39]. These vesicles can be taken up and transfer the intravesicular content into the host intracellular environment, which subsequently modulates cellular activities of the recipient cells. Thus, exosomes provides cells with the ability to signal and transfer the intracellular molecular content within the local microenvironment as well as to distant locations [40].

Recently, it has been found that exosome is an important physiologic vehicle carrying and delivering miRNA. When introduced into a new cytoplasmic space, mature miRNAs associate with the RNA-induced silencing complex to effect gene expression [41]. The selective enrichment of these miRNAs in cellular exosomes, the consistency in expression between different isolations, and the cell-type specificity indicate the presence of a mechanism for their active elaboration within these particles. This may arise from selective transport into a membrane-bound exosome or from sequestration of proteins that are selectively enriched within the exosome. Alternatively, the possibility exists that these miRNAs are rapidly degraded within the cytoplasm, but are protected from degradation when they are sequestered in vesicles before their elaboration from cells as cellular exosomes. Because the tumor cells studied vary in cellular behavior, it is not unexpected that some differences in miRNA content were noted between various cell types [42]. These observations regarding cell-type specificity of miRNA content are similar to those made with respect to protein content of exosomes. They emphasize the need to study and interpret data on individual cell-type basis.

In the present study, a selective set of miRNA was present within EGCG-treated exosomes which was markedly different from that from untreated breast cancer cells. Among the miRNAs by modulated by EGCG, we focused on miR-16 because it had been identified as one of the down-regulated miRNAs in murine and human breast cancer cells. Additionally, targets of miR-16 include many genes related to the control of cell-cycle progression, such as cyclin D1, cyclin E, and the anti-apoptotic protein, Bcl-2 [43-45]. The restoration of miR-16 in prostate cancer cells results in growth arrest, apoptosis and in marked regression of prostate tumor xenografts [45]. A therapeutic strategy is underway that involves the delivery of synthetic miR-16 into advanced prostate tumors [46]. Overexpression of miR-16 was shown to suppress the self-renewal and growth of mouse breast tumor stem cells and to sensitize MCF-7 human breast cancer cells to the chemotherapeutic drug doxorubicin [47]. In addition to the role of tumor suppressor, miR-16 plays a role in macrophages. For example, IKKα mRNA is a target for miR-15 and miR-16. During monocyte-to-macrophage differentiation, a substantial decrease in these miRNAs allows for a considerable increase in IKKα protein and for the subsequent activation of NF-κB pathway [29]. In this study, we have revealed for the first time that EGCG modulates the miRNA profile within tumor exosomes and upregulates miR-16, which was responsible for EGCG-treated exosome down-regulating IKKα and inhibiting M2 polarization of TAM. Considering that miR-16 can function as a tumor suppressor, it is possible that up-regulated exosomal miR-16 might also have had an effect on the survival and proliferation of tumor cells in our *in vivo* experiment.

Macrophage infiltration was decreased by EGCG treatment in the mouse tumor model. To claim that these results are originated from the EGCG-mediated inhibition of macrophage recruitment and M2 polarization rather than the effect of EGCG on macrophage proliferation, we treated TAM isolated from mouse with exosome from EGCG-treated 4T1 cell lines. In these *ex vivo* experiments, we did not observe any decrease in macrophage numbers. However, the mobility of macrophage (data not shown) and the change of phenotype toward non-M2-like macrophages were found to be decreased.

It has been known that NF-κB activation is important for macrophage-to-M2 macrophage differentiation and contributes to tumor progression. Therefore NF-κB in TAM is considered as a novel therapeutic target for cancer control. However, several studies have recently suggested that NF-κB in TAM could have a more complicated and multifaceted role during tumor initiation and progression [48]. For example, in an established murine fibrosarcoma model, the maintenance of M2-phenotype was associated with defective NF-κB activation. This paradoxical role of NF-κB in TAM requires further investigation, and should be addressed in a tumor model-specific manner. The *ex vivo* experiments, using macrophages from established murine breast tumor instead of blood monocytes, would reflect the effect of EGCG on TAM during tumor progression rather than tumor initiation. However for the *in vivo* experiment, EGCG was administrated at the early period after tumor transplantation. We observed the down-regulation of NF-κB pathway in terms of IKKα and I-κB expression in TAM by EGCG or EGCG-treated exosomes *in vivo* and *ex vivo*, which we think would be the molecular mechanism underling the EGCG-mediated hindrance of infiltration and differentiation of macrophages into tumor-promoting M2 macrophages, although direct evidences are lacking.

EGCG has been reported to have anticancer biological activity as well. The main mechanism of this activity involves the inhibition of cell proliferation and induction of apoptosis [34,35]. EGCG can inhibit the cellular proliferation in skin cancer, lung cancer, oral cancer, gastric cancer, intestinal cancer, colon cancer, hepatocellular carcinoma, pancreatic cancer, rectal cancer, prostate cancer and breast cancer, suggesting that EGCG could be utilized as a potential anti-cancer drug [16,36,37]. However, regulation of exosomal miRNAs by EGCG in tumor cells has not previously been studied. We sought to evaluate the ability of breast cancer cells to release vesicles capable of modulating immune response and to investigate modulation of these vesicles by treatment with EGCG. In the present study, we showed that EGCG can modulate the miRNA contained within exosomes and suppresses immune response, and particularly tumor-associated macrophages. The significance of the experiments in this study is that the mechanism by which EGCG mediates communication between the tumor cells and immune cells has been revealed for the first time. Whether this scenario is applicable to other tumors remains to be elucidated.

## Conclusions

In this study, we demonstrated that EGCG can suppress tumor growth through the inhibition of TAM infiltration and M2 polarization, using *in vivo* and *ex vivo* murine breast cancer model. Moreover, we revealed that EGCG modulates miRNAs, particularly up-regulates miR-16, which is transferred to adjacent tumor cells and TAM via tumor-derived exosomes and which has an influence on macrophages in tumor microenvironment.

## Abbreviations

EGCG: Epigallocatechin gallate; TAM: Tumor-associated macrophages.

## Competing interests

The authors declare that they have no other competing interests.

## Authors’ contributions

J-YJ and J-KL have performed the majority of the experiments and were responsible for generating the results, the data analysis, and preparing the manuscript. Y-KJ was responsible for data analysis and for writing the paper. C-WK contributed to the design of the project, data analysis, and writing the manuscript. All authors have reviewed and agreed to the content of the final, submitted version of the manuscript.

## Pre-publication history

The pre-publication history for this paper can be accessed here:

http://www.biomedcentral.com/1471-2407/13/421/prepub

## Supplementary Material

Additional file 1 **4T1 cells were incubated with EGCG (100 μM) and simultaneously (24 hours) transfected with scramble or miR-16 inhibitor for 24 hours.** Exosomes were then extracted from each tumor cells, and total RNA from these cells were extracted and subjected to RT-qPCR to measure the miR-16 level. Histogram shows the relative expression of miR-16 compared to U6 as an internal control. Click here for file

Additional file 2 **RAW264.7 cells were stimulated with 5 μg/ml LPS in the transfection scramble or miR-16 mimics, and total cellular RNA was extracted and submitted to RT-qPCR analysis for IL-1β and IL-6.** Histogram shows the relative expression of molecules compared to GAPDH as internal control. Click here for file

## References

[B1] Gezgen G Roach EC Kizilarslanoglu MC Petekkaya I Altundag K Metabolic syndrome and breast cancer: an overview J Buon 2012 17 223 229 22740197

[B2] Kelsey JL Gammon MD The epidemiology of breast cancer CA Cancer J Clin 1991 41 146 165 1902137 10.3322/canjclin.41.3.146

[B3] Key TJ Schatzkin A Willett WC Allen NE Spencer EA Travis RC Diet, nutrition and the prevention of cancer Public Health Nutr 2004 7 187 200 14972060 10.1079/phn2003588

[B4] Yang CS Sang S Lambert JD Hou Z Ju J Lu G Possible mechanisms of the cancer-preventive activities of green tea Mol Nutr Food Res 2006 50 170 175 16425280 10.1002/mnfr.200500105

[B5] Mukhtar H Ahmad N Tea polyphenols: prevention of cancer and optimizing health Am J Clin Nutr 2000 71 1698S 1702S 10837321 10.1093/ajcn/71.6.1698S

[B6] Lambert JD Hong J Yang GY Liao J Yang CS Inhibition of carcinogenesis by polyphenols: evidence from laboratory investigations Am J Clin Nutr 2005 81 284S 291S 15640492 10.1093/ajcn/81.1.284S

[B7] Stuart EC Scandlyn MJ Rosengren RJ Role of epigallocatechin gallate (EGCG) in the treatment of breast and prostate cancer Life Sci 2006 2006 79 2329 2336 16945390 10.1016/j.lfs.2006.07.036

[B8] Zhang G Miura Y Yagasaki K Effects of green, oolong and black teas and related components on the proliferation and invasion of hepatoma cells in culture Cytotechnology 1999 31 37 44 19003122 10.1023/A:1008076306672 PMC3449775

[B9] Lu G Liao J Yang G Reuhl KR Hao X Yang CS Inhibition of adenoma progression to adenocarcinoma in a 4-(methylnitrosamino)-1-(3-pyridyl)-1-butanone-induced lung tumorigenesis model in A/J mice by tea polyphenols and caffeine Cancer Res 2006 66 11494 11501 17145898 10.1158/0008-5472.CAN-06-1497

[B10] Lu YP Lou YR Xie JG Topical applications of caffeine or (−)-epigallocatechin gallate (EGCG) inhibit carcinogenesis and selectively increase apoptosis in UVB-induced skin tumors in mice Proc Natl Acad Sci U S A 2002 99 12455 12460 12205293 10.1073/pnas.182429899 PMC129466

[B11] Thangapazham RL Singh AK Sharma A Warren J Gaddipati JP Maheshwari RK Green tea polyphenols and its constituent epigallocatechin gallate inhibits proliferation of human breast cancer cells *in vitro* and *in vivo* Cancer Lett 2007 245 232 241 16519995 10.1016/j.canlet.2006.01.027

[B12] Adhami VM Siddiqui IA Ahmad N Gupta S Mukhtar H Oral consumption of green tea polyphenols inhibits insulin-like growth factor-I-induced signaling in an autochthonous mouse model of prostate cancer Cancer Res 2004 64 8715 8722 15574782 10.1158/0008-5472.CAN-04-2840

[B13] Wang ZY Huang MT Ho CT Inhibitory effect of green tea on the growth of established skin papillomas in mice Cancer Res 1999 52 6657 6665 1423310

[B14] Heijnen HF Schiel AE Fijnheer R Activated platelets release two types of membrane vesicles: microvesicles by surface shedding and exosomes derived from exocytosis of multivesicular bodies and alphagranules Blood 1999 94 3791 3799 10572093

[B15] Rozmyslowicz T Majka M Kijowski J Platelet- and megakaryocytederived microparticles transfer CXCR4 receptor to CXCR4-null cells and make them susceptible to infection by X4-HIV AIDS 2003 17 33 42 12478067 10.1097/00002030-200301030-00006

[B16] Majka M Janowska-Wieczorek A Ratajczak J Numerous growth factors, cytokines, and chemokines are secreted by human CD34(+) cells, myeloblasts, erythroblasts, and megakaryoblasts and regulate normal hematopoiesis in an autocrine/paracrine manner Blood 2001 97 3075 3085 11342433 10.1182/blood.v97.10.3075

[B17] Kim HK Song KS Park YS Kang YH Lee YJ Lee KR Kim HK Ryu KW Bae JM Kim S Elevated levels of circulating platelet microparticles, VEGF, IL-6 and RANTES in patients with gastric cancer: possible role of a metastasis predictor Eur J Cancer 2003 39 184 191 12509950 10.1016/s0959-8049(02)00596-8

[B18] Iero M Valenti R Huber V Filipazzi P Parmiani G Fais S Rivoltini L Tumour-released exosomes and their implications in cancer immunity Cell Death Differ 2008 15 80 88 17932500 10.1038/sj.cdd.4402237

[B19] Safaei R Larson BJ Cheng TC Gibson MA Otani S Naerdemann W Howell SB Abnormal lysosomal trafficking and enhanced exosomal export of cisplatin in drug-resistant human ovarian carcinoma cells Mol Cancer Ther 2005 4 1595 1604 16227410 10.1158/1535-7163.MCT-05-0102

[B20] Millimaggi D Mari M D'Ascenzo S Carosa E Jannini EA Zucker S Carta G Pavan A Dolo V Tumor vesicle-associated CD147 modulates the angiogenic capability of endothelial cells Neoplasia 2007 9 349 357 17460779 10.1593/neo.07133 PMC1854851

[B21] Camussi G Deregibus MC Bruno S Grange C Fonsato V Tetta C Exosome/microvesicle-mediated epigenetic reprogramming of cells Am J Cancer Res 2011 1 98 110 21969178 PMC3180104

[B22] Pollard JW Macrophages define the invasive microenvironment in breast cancer J Leukoc Biol 2008 84 623 630 18467655 10.1189/jlb.1107762 PMC2516896

[B23] Bingle L Brown NJ Lewis CE The role of tumor-associated macrophages in tumor progression: implications for new anticancer therapies J Pathol 2002 196 254 265 11857487 10.1002/path.1027

[B24] Leek RD Harris AL Tumor-associated macrophages in breast cancer J Mammary Gland Biol Neoplasia 2002 7 177 189 12463738 10.1023/a:1020304003704

[B25] Antonio S Tiziana S Alberto M Paola A Tumour-associated macrophages are a distinct M2 polarised population promoting tumour progression: Potential targets of anti-cancer therapy Eur J Cancer 2006 42 717 727 16520032 10.1016/j.ejca.2006.01.003

[B26] Saccani A Schioppa T Porta C Biswas SK Nebuloni M Vago L Bottazzi B Colombo MP Mantovani A Sica A p50 nuclear factor-κB overexpression in tumor-associated macrophages inhibits M1 inflammatory responses and antitumor resistance Cancer Res 2006 66 11432 11440 17145890 10.1158/0008-5472.CAN-06-1867

[B27] Mantovani A Marchesi F Porta C Sica A Allavena P Inflammation and cancer: breast cancer as a prototype Breast 2007 16 S27 S33 17764938 10.1016/j.breast.2007.07.013

[B28] Paik S Shak S Tang G A multigene assay to predict recurrence of tamoxifen-treated, node-negative breast cancer N Engl J Med 2004 351 2817 2826 15591335 10.1056/NEJMoa041588

[B29] Li T Morgan MJ Choksi S Zhang Y Kim YS Liu ZG MicroRNAs modulate the noncanonical NF-κB pathway by regulating IKKα expression during macrophage differentiation Nat Immunol 2010 11 799 805 20711193 10.1038/ni.1918 PMC2926307

[B30] Hagemann T Lawrence T McNeish I Charles KA Kulbe H Thompson RG Robinson SC Balkwill FR Re-educating tumor-associated macrophages by targeting NF-kappaB J Exp Med 2008 205 1261 1268 18490490 10.1084/jem.20080108 PMC2413024

[B31] Shih JY Jeremy AY Chen JW Yang PC Tumor-associated macrophage: its role in cancer invasion and metastasis J Cancer Mol 2006 2 101 106

[B32] Bobrie A Colombo M Krumeich S Raposo G Théry C Diverse subpopulations of vesicles secreted by different intracellular mechanisms are present in exosome preparations obtained by differential ultracentrifugation J Extracellular Vesicles 2012 1 18397 10.3402/jev.v1i0.18397 PMC3760636 24009879

[B33] Chew V Toh HC Abastado JP Immune microenvironment in tumor progression: characteristics and challenges for therapy J Oncol 2012 2012 1 10 10.1155/2012/608406 PMC3423944 22927846

[B34] Théry C Exosomes: secreted vesicles and intercellular communications F1000 Biology Reports 2011 3 15 21876726 10.3410/B3-15 PMC3155154

[B35] Camussi G Deregibus MC Bruno S Cantaluppi V Biancone L Exosomes/microvesicles as a mechanism of cell-to-cell communication Kidney Int 2010 78 838 848 20703216 10.1038/ki.2010.278

[B36] Rustom A Saffrich R Markovic I Nanotubular highways for intercellular organelle transport Science 2004 303 1007 1010 14963329 10.1126/science.1093133

[B37] Sherer NM Mothes W Cytonemes and tunnelling nanotubules in cell-cell communication and viral pathogenesis Trends Cell Biol 2008 18 414 420 18703335 10.1016/j.tcb.2008.07.003 PMC2628975

[B38] Ratajczak J Wysoczynski M Hayek F Membrane-derived microvesicles: important and underappreciated mediators of cell-to-cell communication Leukemia 2006 20 1487 1495 16791265 10.1038/sj.leu.2404296

[B39] Hutagalung AH Novick PJ Role of Rab GTPases in membrane traffic and cell physiology Physiol Rev 2011 91 119 149 21248164 10.1152/physrev.00059.2009 PMC3710122

[B40] Skog J Wurdinger T van Rijn S Meijer DH Gainche L Sena-Esteves M Glioblastoma microvesicles transport RNA and proteins that promote tumour growth and provide diagnostic biomarkers Nat Cell Biol 2008 10 1470 1476 19011622 10.1038/ncb1800 PMC3423894

[B41] Pillai RS MicroRNA function: multiple mechanisms for a tiny RNA? RNA 2005 11 1753 1761 16314451 10.1261/rna.2248605 PMC1370863

[B42] Pigati L Yaddanapudi SC Iyengar R Kim DJ Hearn SA Danforth D Hastings ML Duelli DM Selective release of MicroRNA species from normal and malignant mammary epithelial cells PLoS One 2010 5 e13515 20976003 10.1371/journal.pone.0013515 PMC2958125

[B43] Liu Q Fu H Sun F Zhang H Tie Y Zhu J Xing R Sun Z Zheng X miR-16 family induces cell cycle arrest by regulating multiple cell cycle genes Nucleic Acids Res 2008 36 5391 5404 18701644 10.1093/nar/gkn522 PMC2532718

[B44] Ofir M Hacohen D Ginsberg D miR-15 and miR-16 are direct transcriptional targets of E2F1 that limit E2F-induced proliferation by targeting cyclin E Mol Cancer Res 2011 9 440 447 21454377 10.1158/1541-7786.MCR-10-0344

[B45] Bonci D Coppola V Musumeci M Addario A Giuffrida R Memeo L D’Urso L Pagliuca A Biffoni M Labbaye C Bartucci M Muto G Peschle C De Maria R The miR-15a-miR-16-1 cluster controls prostate cancer by targeting multiple oncogenic activities Nat Med 2008 14 1271 1277 18931683 10.1038/nm.1880

[B46] Takeshita F Patrawala L Osaki M Takahashi RU Yamamoto Y Kosaka N Kawamata M Kelnar K Bader AG Brown D Ochiya T Systemic delivery of synthetic microRNA-16 inhibits the growth of metastatic prostate tumors via downregulation of multiple cell-cycle genes Mol Ther 2010 18 181 187 19738602 10.1038/mt.2009.207 PMC2839211

[B47] Zhang X Wan G Mlotshwa S Vance V Berger FG Chen H Lu X Oncogenic Wip1 phosphatase is inhibited by miR-16 in the DNA damage signaling pathway Cancer Res 2010 70 7176 7186 20668064 10.1158/0008-5472.CAN-10-0697 PMC2940956

[B48] Thorsten H Subhra KB Toby L Antonio S Claire EL Regulation of macrophage function in tumors: the multifaceted role of NF-κB Blood 2009 113 3139 3146 19171876 10.1182/blood-2008-12-172825 PMC2869029

