# Supplementary figures and images for: Exosome derived from epigallocatechin gallate treated breast cancer cells suppresses tumor growth by inhibiting tumor-associated macrophage infiltration and M2 polarization
Source: BMC Cancer. 2013 Sep 17;13:421. doi: 10.1186/1471-2407-13-421 (PMC3848851; doi:10.1186/1471-2407-13-421)

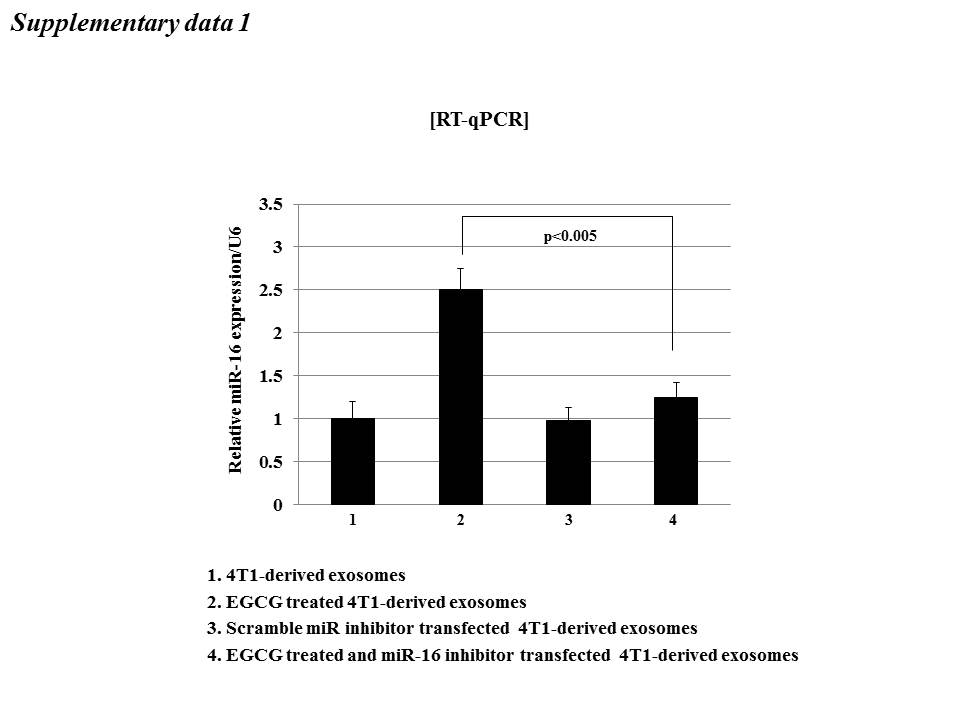

Supplement: Additional file 1 — 4T1 cells were incubated with EGCG (100 μM) and simultaneously (24 hours) transfected with scramble or miR-16 inhibitor for 24 hours. Exosomes were then extracted from each tumor cells, and total RNA from these cells were extracted and subjected to RT-qPCR to measure the miR-16 level. Histogram shows the relative expression of miR-16 compared to U6 as an internal control. [file 1471-2407-13-421-S1.jpeg]

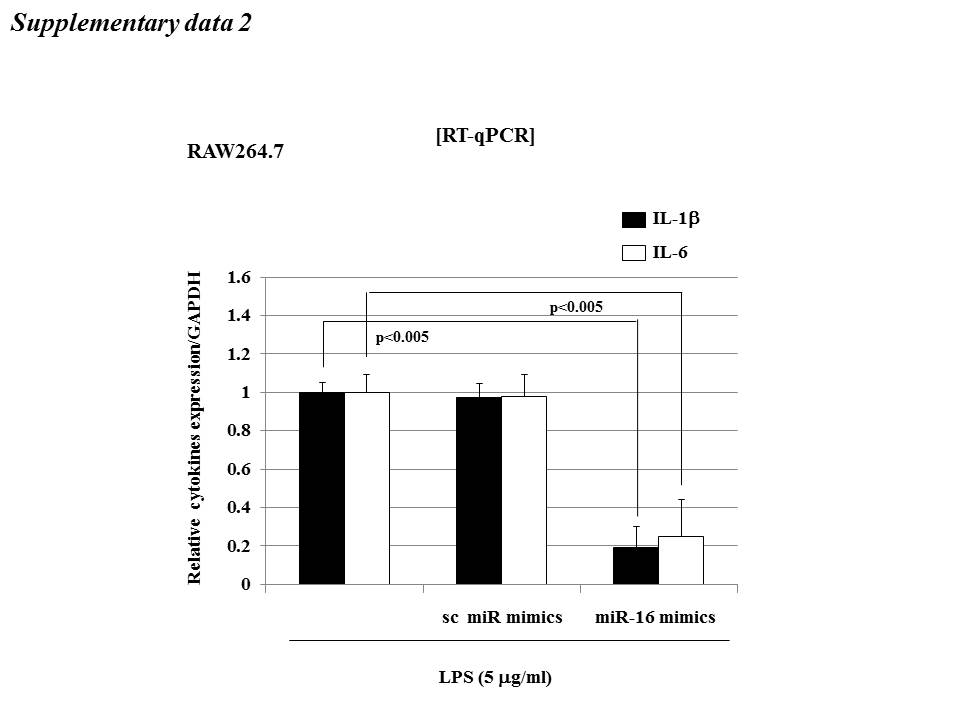

Supplement: Additional file 2 — RAW264.7 cells were stimulated with 5 μg/ml LPS in the transfection scramble or miR-16 mimics, and total cellular RNA was extracted and submitted to RT-qPCR analysis for IL-1β and IL-6. Histogram shows the relative expression of molecules compared to GAPDH as internal control. [file 1471-2407-13-421-S2.jpeg]
